# Use of Adjuvanted Quadrivalent Influenza Vaccine in Older-Age Adults: A Systematic Review of Economic Evidence

**DOI:** 10.3390/vaccines12050523

**Published:** 2024-05-10

**Authors:** Ciaran O’Neill, Grainne E. Crealey

**Affiliations:** 1 Centre for Public Health, Institute of Clinical Sciences, Royal Victoria Hospital, Belfast BT12 6BA, UK; 2 Clinical Costing Solutions, Belfast BT15 4EB, UK; grainnecrealey@gmail.com

**Keywords:** influenza, vaccination, cost-effectiveness, older persons, systematic review

## Abstract

Influenza vaccination is an important public health measure that can reduce disease burden, especially among older persons (those aged 65 and over) who have weaker immune systems. Evidence suggests enhanced vaccines, including adjuvanted quadrivalent vaccines (aQIV), may be particularly effective in this group. This study reports the results of a systematic review of the cost-effectiveness of aQIV in this population. The review was undertaken and reported in accordance with good practice guidelines. Medline and EMBASE were searched from 2013 to the present. Pre-selected eligibility criteria were employed and quality assessment undertaken using the Consensus Health Economic Criteria (CHEC-extended) checklist and Consolidated Health Economic Evaluation Reporting Standard (CHEERS) 2022 checklists. A total of 124 records were returned, with 10 full text papers retained. All were modelling studies and exhibited heterogeneity in approach, perspective, and parameter estimation. Nine papers reported cost-effectiveness ranging from EUR 6694/QALY to EUR 20,000/QALY in evaluations employing a payer perspective and from EUR 3936/QALY to EUR 17,200/QALY in those using a societal perspective. Results remained robust to a range of sensitivity analyses. One paper that reported contrary findings adopted a distinct modelling approach. It is reasonable to conclude that there is a broad consensus as to the cost-effectiveness of aQIV in this population group.

## 1. Introduction

Seasonal influenza, or the flu, is a common and potentially serious respiratory infection caused by influenza viruses. It affects about a billion people every year, causing 3–5 million cases of severe illness and 290,000 to 650,000 deaths, mostly in developing countries [1]. Seasonal influenza also imposes a significant economic burden on society, as it leads to increased healthcare costs, absenteeism from work, and reduced productivity [2]. According to one study, the annual cost of influenza in the United States alone was estimated at USD 87.1 billion in 2015 [3]. Another study found that the economic impact of influenza in those aged 65 years and older ranged from USD 1.6 billion to USD 5.2 billion per year in the USA, and from EUR 0.2 billion to EUR 1.3 billion per year in Europe [4]. Therefore, seasonal influenza is not only a major public health challenge but also a socio-economic problem that requires effective prevention and control measures.

Vaccination against influenza is an important public health measure to protect vulnerable populations, especially older persons. Influenza can cause serious complications, such as pneumonia, hospitalization, and even death for older adults who have weaker immune systems. According to a 2018 study, flu vaccination among adults reduced the risk of being admitted to an ICU with the flu by 82% [5]. Another study found that flu vaccines reduced the risk of flu-associated hospitalization among older adults by about 40% on average from 2009 to 2016 [6]. A 2017 study also reported that well-matched vaccines provided benefits in reducing related admissions to hospital and pneumonia for elderly individuals living in the community [7].

A range of vaccination options is available, including trivalent (TIV) and quadrivalent (QIV) inactivated vaccines, adjuvanted TIV (aTIV), high-dose TIV (hdTIV), intradermal TIV (idTIV), cell culture-derived QIV (ccQIV), and recombinant QIV (rQIV) [8]. QIV appears to offer additional protection compared to TIV [9], containing antigens derived from an additional influenza type B virus. However, among older persons, standard-dose influenza vaccines like TIV and QIV have suboptimal immunogenicity and efficacy [7,10] owing to the phenomenon of immunosenescence and a relatively high prevalence of chronic conditions, including multi-morbidity, among older adults. To augment immune responses, enhanced vaccines use different strategies—high-dose influenza vaccines (high-dose TIV or high-dose QIV) contain four times more hemagglutinin antigen than standard-dose vaccines, whereas adjuvanted vaccines (aTIV or aQIV) contain the adjuvant MF59, which has been shown to increase the magnitude and breadth of immune responses [11].

The increasing importance of demonstrating value for money by payers means reimbursement decisions cannot, however, be based on an assessment of efficacy alone. This has increased interest in systematic reviews of cost and cost-effectiveness outcomes (SR-CCEOs). Context is, however, important to the outcome of evaluations; local healthcare costs and productivity losses, for example, can vary widely and so too can the conclusions of evaluations independent of efficacy data. However, whilst systematic reviews and meta-analyses of clinical efficacy data can often be readily synthesised to produce rigorous evidence to support decision-makers, it is more challenging to combine findings from economic evaluations, as there is frequently a range of sources of heterogeneity in applied methods and reporting,

For instance, a systematic review of cost-effectiveness studies on replacing TIV with QIV has established that the incremental cost–utility ratio (ICUR) of QIV vs. TIV ranged from likely not cost-effective (≥USD 140,000 per quality-adjusted life year (QALY) gained) to cost-saving [8].

For a variety of reasons—both ethical and practical—there is a lack of real-world evidence (RWE) on the cost-effectiveness of alternative vaccines derived from clinical trials. Because of this, evidence of cost-effectiveness has typically been derived from modelling studies. Assessing such evidence requires an assessment of the comparators, the models, and—given the importance of assumptions made in studies to their outcomes—of the sensitivity analyses to which models are subjected.

The aim of this SR-CCEO is to assess evidence of the cost-effectiveness of aQIV in the context of influenza prevention among those aged 65 and over. We examine aQIV specifically given the emergence of a growing body of literature relating to its cost-effectiveness in this population and the needs of decision-makers to optimise resource allocation and to enhance patient outcomes.

## 2. Methods

The International Society for Pharmacoeconomic Outcomes and Research (ISPOR) good practice task force report for the critical appraisal of systematic reviews with costs and cost-effectiveness outcomes (SR-CCEOs) was used to inform the conduct of this review [12], and findings are reported in accordance with the Preferred Reporting Items for Systematic Reviews and Meta-Analyses (PRISMA) statement [13]. While a protocol was prepared prior to conduct of the analysis, it was not submitted for publication to Prospero, as data extraction commenced earlier than had been anticipated.

### 2.1. Eligibility Criteria

A systematic literature review was performed to identify economic evaluations of adjuvanted quadrivalent influenza vaccines (aQIV) in older adults. The PICO tool (Population, Intervention, Comparator, Outcomes) provided a framework for development of the research question. Primary studies of interest were those that provided a comparative analysis in terms of cost and outcomes of aQIV relative to an identified alternative. Studies were likely to be model-based and full economic evaluations (e.g., cost-effectiveness (CEA), cost–utility (CUA), budget impact (BIA), and cost–benefit analyses (CBA)), though data from randomised controlled trials were eligible. Studies were included if participants were aged 65 years or older. No restrictions were imposed with regard to risk profile (e.g., at low or high risk of developing influenza-related complications) or setting (e.g., community dwelling or nursing home residents). Eligibility for inclusion was limited to original peer-reviewed research papers (i.e., not abstracts, letters to the editor, commentaries, editorials, conference proceedings, or reviews) published in English.

### 2.2. Search Strategy

Medline (via OVID), EMBASE (via OVID, 1974–December 2023), and ECONLIT were searched from 2013 to 14 December 2023 during the time of preparation. This time qualifier was imposed to ensure the relevance of records included in the review. Search terms were identified to capture older adults (≥65 years), as was a range of economic evaluation modalities. For the purpose of increasing sensitivity, only terms relating to adjuvanted quadrivalent influenza vaccines were searched for (Supplementary Table S1). Reference lists of retrieved studies were reviewed.

### 2.3. Handling Searches

A PRISMA (Preferred Reporting Items for Systematic Reviews and Meta-Analyses) flow chart was used to document study selection, illustrating the numbers of records retrieved and selection flow through the screening rounds; all excluded records (with rationale for exclusion) were documented.

### 2.4. Selection of Studies

Two screening rounds were conducted independently by two health economists experienced in undertaking reviews (CON, GC). The first round screened the title and abstract of the articles based on the eligibility criteria; those selected at this stage entered a second round of full-text screening with eligibility based on the inclusion and exclusion criteria. Any disagreements were discussed among the two reviewers, with recourse to a third reviewer if necessary.

### 2.5. Data Extraction and Management

Data were extracted by both reviewers using a proforma based on Winjen et al. (2016) [14]. A range of information was extracted relating to the study design and setting, type of economic evaluation, and model parameters. Quality assessment, key findings (such as the drivers of cost-effectiveness), and author conclusions were extracted. The data extraction form was piloted by two reviewers (GC and CON) on one paper and discussion was used to ensure consistent application thereafter.

### 2.6. Assessment of Study Quality

Two reviewers (CON, GC) independently assessed study quality. Full economic evaluations were assessed using the Consensus Health Economic Criteria (CHEC-extended) checklist [15], which is designed to be used as a screening tool to identify studies that meet a minimum quality standard for inclusion in a systematic review. The CHEERS (2022) checklist was used to assess the quality of reporting [16].

### 2.7. Data Synthesis

**Figure 1:**
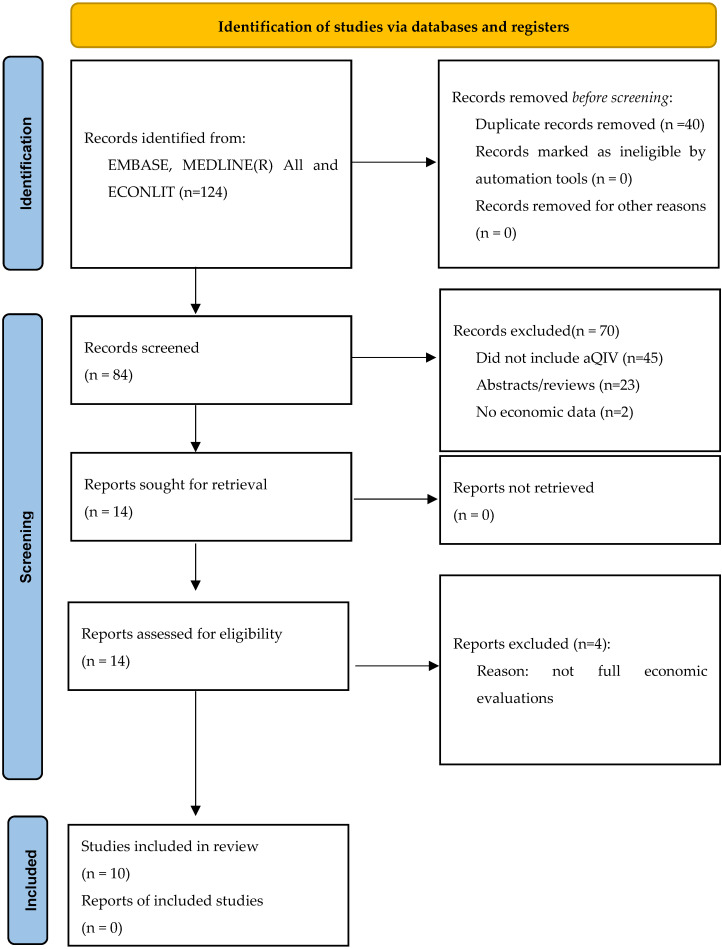
The Preferred Reporting Items for Systematic Reviews and Meta-Analyses (PRISMA) flow diagram of study selection.

Heterogeneity in data was explored, and Figure 1 of the ISPOR Good Practices Task Force Report [12] (was used to determine the most appropriate approach to data synthesis. Study characteristics and extracted data were summarised in text and tabulated. A formal meta-analysis was not possible given the degree of heterogeneity among the published studies, and a graphical presentation of mean incremental cost-effectiveness ratios (ICERs) along with their distribution was not, in consequence, possible. The key aspects assessed included the reporting of key information (e.g., study design and how parameters were identified), the modelling methods used, and the validity of cost and health benefit estimates.

## 3. Results

### 3.1. Systematic Search

Database searches returned 124 records; from this, 40 duplicates were removed, leaving 84 reports. Fourteen papers were assessed against the inclusion and exclusion criteria, resulting in 10 full-text papers for inclusion in the review [17,18,19,20,21,22,23,24,25,26]. The PRISMA 2020 diagram is presented in Figure 1.

### 3.2. Characteristics of Included Studies

The main characteristics of the included studies are contained in Table 1. All studies (as per the eligibility criteria) were in English and involved the comparison of an adjuvanted quadrivalent influenza vaccine against a relevant comparator in a population of older persons. All studies were conducted in high-income countries, namely, Italy [18,26], Spain [19,21,25], Norway, Denmark, Sweden [22], the United Kingdom [17], Germany [20], Belgium [23], and Ireland [24], and all studies were funded/supported by a pharmaceutical company and/or included industry employees as authors.

The study population was individuals aged 65 years and older for all studies. All studies stratified this population further into age categories (e.g., 65–74 years, 75+ years etc.), allowing differential risk of complications with increasing age to be examined. For some of the analyses [17,18,19,20,24], influenza transmission was simulated using a deterministic Susceptible–Exposed–Infectious–Removed (SEIR) model to calculate attack rates of confirmed seasonal influenza infection by age group and viral subtype over the seasons considered. Models adopting this approach included all age groups (accounting for “herd immunity”), as opposed to models that included only the population under consideration [21,22,23,25,26].

Three studies compared aQIV with standard-dose QIV [18,19,24], three papers compared aQIV to high-dose QIV [17,21,26], three compared aQIV with both standard-dos QIV and high-dose QIV [20,22,23], and one study compared aQIV with recombinant QIV [25].

Despite the time period chosen for the review (2013–2023), all studies were published between 2021 and 2023. All were full economic evaluations that compared costs and outcomes for at least two comparators. All studies included quality-adjusted life-year (QALY) as an outcome measure, reporting findings in terms of “cost per QALY”. Four papers also reported other outcomes (such as life year gained) [18,21,22,23].

### 3.3. Study Design

Two modelling modalities prevailed in the studies reviewed: static decision tree models and hybrid models that used a dynamic Susceptible–Exposed–Infectious–Recovered (SEIR) model for the epidemiological module that simulated the natural history of the disease to produce estimates of morbidity- and mortality-related complications for use in the static decision tree.

The level of detail provided regarding model structure and rationale for the choice of parameter values used in the models varied and no studies included an open-access model alongside the paper. For example, one paper [18] directed readers to a full health technology assessment (HTA) document published in the Italian language, whereas another [24] referred readers to other published literature sources where information on parameter estimates were available. Kohli et al. (2022) provided a detailed technical appendix. None of the included studies provided information on all parameter estimates used in the model, ranges for sensitivity analysis (including justification for distributions chosen), or references relating to data sources used. However, this observation is not unusual in the current modelling literature.

All but three of the studies employed both a payer and a societal perspective [17,23,26]. The time horizons used in the evaluations were generally 1 year (or one flu season), with data from up to 10 flu seasons being used in 4 studies [17,18,19,20]. All papers reported the discount rate used for both costs and outcomes and cited the rationale for this; in the majority of studies, costs were collected for 12 months or less (time horizon of 1 year or one influenza season), which did not require discounting to be performed; outcomes were discounted over longer time periods (i.e., a lifetime).

Heterogeneity was observed in the model structure, the choice of epidemiological variables included, and the complexity/rigour with which the costs and outcomes associated with influenza complications were estimated. The level of heterogeneity observed suggested that a rigorous narrative synthesis/comparison of the models should be undertaken rather than data synthesis. For example, direct healthcare costs were estimated using a number of approaches. One study [22] presented data on a wide range of influenza-related complications (such as bronchitis, pneumonia, urinary tract infection (URTI), myocarditis, stroke, gastrointestinal bleed, etc.), with detailed information on incidence, cost (inpatient and outpatient), and utility decrements associated with each complication. In another study, economic input parameters were based on estimates from a published study [24]. All studies at a minimum included the cost of general practitioner (GP) visits, outpatient episodes, and vaccination acquisition costs. Some studies reported the method used to account for productivity losses (friction cost or human capital approach). Only one study included productivity losses attributable to carers [21]; no studies stratified absenteeism/productivity loss by influenza subtype.

As mentioned earlier, QALYs were the primary outcome measure used in the studies reviewed. This outcome measure requires data on baseline health-related quality of life (HRQoL) for the population under consideration, decrements in HRQoL associated with the disease/condition, and an estimate of the time spent in that condition (i.e., duration of symptoms). Significant heterogeneity was observed in parameter estimates used in this regard. For example, one study assumed a disutility of 0.15 for 35 days due to symptomatic influenza [23], whereas another study assumed a disutility value for symptomatic outpatients of 0.33 for 7 days and a disutility value for inpatients of 0.6 for 21 days [21]. All studies included QALY loss associated with flu symptoms and/or complications, and all studies included QALY loss associated with influenza-related mortality. Country-specific utility estimates were employed in most cases, and where data were not available, published data from adjacent jurisdictions were used. No studies indicated that the utility values were based on influenza subtypes.

### 3.4. Presentation of Findings

All studies presented findings from the cost–utility analysis as incremental cost-effectiveness ratios (ICERs) and indicated whether this ratio was acceptable relative to the willingness to pay threshold in that jurisdiction (i.e., represented value for money). All studies presented uncertainty in model finding using deterministic sensitivity analysis (DSA) and probabilistic sensitivity analysis (PSA). Seven studies presented the results of deterministic sensitivity analyses (DSA) in a tornado diagram (i.e., a graphical representation of the impact of each parameter on the ICER) [19,20,21,22,23,25,26]. Seven studies presented a scatter plot of the findings on the cost-effectiveness plane [18,19,21,22,23,25,26] and four presented a cost-effectiveness acceptability curve (CEAC) [20,22,23,26].

Quality assessment of the included papers was undertaken using the CHEC-extended checklist [15] and assessment of the quality of reporting was undertaken using the 28-item Consolidated Health Economic Evaluation Reporting Standards checklist (CHEERS 2022) [16] (Supplementary Table S2). All studies exhibited high compliance levels with the CHEERS 2022 checklist, as would be expected given their recent publication dates. Lower compliance was observed for the items that have been recently added to the updated checklist.

However, non- or partial compliance with some established checklist items was observed, such as provision of a health economics plan (item 4), provision of rationale for the model and stating whether the model was publicly available (item 16), reporting model validation procedures (item 17), and reporting of all analytic inputs, including uncertainty or distributional effects (item 22). Compliance with the CHEC checklist was high across all papers.

Findings from the included studies are summarised in Table 2. Details of the key assumptions used in the cost-effectiveness model, key findings (base-case scenario), results of uncertainty analyses, and author conclusions are presented.

#### 3.4.1. Evidence on the Cost-Effectiveness of aQIV versus Standard-Dose QIV

Evidence was presented on the cost-effectiveness of aQIV versus standard-dose QIV in six papers [18,19,20,22,23,24]. In all analyses, from both the payer and the societal perspectives, aQIV was cost-effective compared to standard-dose QIV in this older patient population. Incremental cost-effectiveness ratios (ICERs) ranged from EUR 6694/QALY to EUR 20,000/QALY in evaluations employing a payer perspective and from EUR 3936/QALY to EUR 17,200/QALY in those using a societal perspective. When these base-case findings were subjected to deterministic sensitivity analysis (DSA), a core group of key drivers of cost-effectiveness was identified: relative vaccine effectiveness, relative vaccine cost, influenza attack rate, and vaccine coverage. When subjected to probabilistic sensitivity analysis (PSA), where model inputs are varied simultaneously, aQIV was deemed cost-effective in between 52.4% and 95% of iterations relative to the willingness-to-pay threshold for each country.

#### 3.4.2. Evidence on the Cost-Effectiveness of aQIV versus High-Dose QIV

Evidence was presented on the cost-effectiveness of aQIV versus high-dose QIV in six papers [17,20,21,22,23,26]. In all analyses except one [26], aQIV was cost-saving; this finding was driven by the similarity in vaccine effectiveness and lower cost of aQIV. The opposite was reported by Rumi et al. (2023). In the absence of head-to-head clinical trials comparing high-dose QIV and aQIV, the effectiveness of high-dose QIV versus aQIV was calculated indirectly based on the relative efficacy of each vaccine versus a common comparator: standard dose (standard-dose QIV). For this calculation, they therefore assumed that the relative efficacy of aQIV vs. standard-dose QIV in the base-case analysis was 0% and that the relative efficacy of high-dose QIV vs. standard-dose QIV was 24.2%.

#### 3.4.3. Evidence on the Cost-Effectiveness of aQIV Compared to Recombinant QIV

Only one paper compared aQIV with recombinant QIV [25]. In this evaluation, aQIV was more cost-effective than recombinant QIV in 99.7% of simulations at a willingness-to-pay threshold of EUR 25,000/QALY. Vaccine cost and vaccine coverage were key drivers of cost-effectiveness.

## 4. Discussion

This paper is the first systematic review to assess evidence of the cost-effectiveness of aQIV in an older population. Across the ten papers identified, six studies examined the cost-effectiveness of aQIV versus standard-dose QIV, six examined the cost-effectiveness of aQIV versus high-dose QIV, and one paper examined the cost-effectiveness of aQIV and QIVr. Nine papers concluded that aQIV was cost-effective against its comparator (when compared to standard-dose QIV or QIVr and cost-saving when compared to high-dose QIV). These findings were robust when subjected to sensitivity analyses, suggesting the likelihood of cost-effectiveness is high even in the face of parameter uncertainty. The main factors that influenced cost-effectiveness were the relative effectiveness and cost of the vaccines, the influenza infection rate, and the vaccine coverage. That differences in relative vaccine effectiveness and vaccine acquisition price may drive differences in cost-effectiveness estimates has been reported elsewhere [9,11], and the influence of these variables on cost-effectiveness estimates is reflected in the World Health Organization (WHO) recommendation that sensitivity analyses for economic evaluations should be performed on five key parameters. These are discount rates, vaccine efficacy, influenza incidence, influenza complication rates, and vaccine price [27]. Furthermore, when relative vaccine efficacy estimates for aTIV/aQIV and high-dose TIV/high-dose QIV are comparable, the vaccine acquisition price can be the main driver of cost-effectiveness estimates [11]. Under these circumstances, robust comparative assessment is challenging, as the acquisition price of vaccines is not always transparent due to proprietary negotiation and rebates. However, to address this, some studies have employed adjustment methods to estimate acquisition and administration costs [27,28]. A number of other factors were also identified during critical appraisal of the papers (using the CHEC-checklist) and assessment of the quality of reporting of studies (using the CHEERS 2022 checklist).

One such factor was the choice of modelling approach used. Dynamic transmission models can account for changes in the incidence of the disease over time, the interactions between vaccinated and unvaccinated individuals, and the potential emergence of new variants of the disease. Such complex models are extremely valuable in infectious disease modelling, as they can incorporate varying disease state disutility inputs, likelihood of transition between different disease states, and duration of disease [29,30]. These models have feedback loops, as the probability of a susceptible individual becoming infected at any one point in time (the force of infection) is related to the number of infections in the population and will change over time and feed back into the future force of infection [31]. Static models use a fixed rate of infection and do not measure the indirect impact of vaccination, such as herd immunity. Half of the studies reviewed employed a hybrid approach, using dynamic modelling for the epidemiological compartment of the model paired with a static decision tree. The impact of dynamic models over static models will be more pronounced in countries with universal vaccination compared to countries with influenza vaccination restricted to elderly and high-risk groups [9]. Hence, this may lead to underestimating the total savings from vaccination, but it does not change the main conclusions of this review.

Cost-effectiveness data can inform health policy and resource allocation decisions, but they require transparent and rigorous modelling. However, none of the studies we reviewed shared their models openly, which limits the validity and applicability of their findings. This is a common problem in both academic and industry settings, where code-sharing is not yet incentivised [31,32]. We also found that the quality of reporting was inconsistent across studies, especially regarding the justification and precision of the model parameters. For example, some studies did not report in detail how they estimated the healthcare costs of influenza complications or productivity losses, and the measurement of influenza morbidity was frequently based on older studies or using data from different jurisdictions.

More complex models may not necessarily improve the cost-effectiveness estimates of aQIV, as they still depend on assumptions to fill the gaps in the existing literature. Therefore, we suggest using real-world evidence (RWE) to address these gaps and provide more reliable data. Postma and colleagues (2023) provide a thorough overview of the many issues relating to the use of RWE in cost-effectiveness analyses of enhanced influenza vaccines in older adults [11]. Additionally, we recommend making the models open-access for the purposes of transparency and so they can be updated or recalculated as new or revised data become available. An example of an open-access economic model can be found in Chit et al. (2015) [33].

Two points of note: First, as the number of circulating strains can vary over time, the relative cost-effectiveness of trivalent and quadrivalent vaccines may vary. While formally outside the scope of this review, it has been shown that enhanced trivalent influenza vaccines (aTIV and high-dose TIV) are more effective compared with standard-dose vaccines (standard-dose TIV) in preventing influenza-related hospitalisations and deaths in older adults. Several systematic reviews have assessed the economic value of aTIVs in this population using different methods, perspectives, and settings [8,11,30,34,35,36]. The results vary depending on the assumptions and parameters used, but most studies suggest that aTIVs are cost-effective or cost-saving compared to other standard-dose influenza vaccines. As with aQIV, differences in relative vaccine effectiveness estimates and acquisition price can drive differences in cost-effectiveness estimates between enhanced vaccines, especially in seasons with a poor vaccine match or a high disease burden. Similar to our findings with respect to the cost-effectiveness of aQIV, factors that influence the cost-effectiveness of aTIVs include vaccine price, vaccine uptake, vaccine effectiveness, influenza incidence, and healthcare costs associated with influenza complications. However, it should be noted that there is a lack of head-to-head comparisons between QIV, high-dose TIV, and aTIV. Furthermore, owing to influenza virus mutations and the potential for vaccine mismatch data used to populate cost-effectiveness models, data should be drawn from multiple influenza seasons. The disappearance of the B Yamagata strain over the past four seasons, for example, may be material in future considerations in this regard. Second, access to vaccines may well impact uptake, which in turn may impact the risk of reinfection as well as the equity of health outcomes. Where access to aQIV is dependent on the ability to pay, its effectiveness and cost-effectiveness may be adversely impacted and health inequalities widened. These factors warrant consideration as part of the broader issues that may feature in a health technology assessment of the intervention.

A possible perceived weakness of this study is the industry involvement in funding or sponsoring all the included studies. This is not uncommon in this field, as shown by other systematic reviews [8,9]. However, this could raise doubts about the study outcomes, as the sponsors might favour results that benefit their products. This underscores our recommendation that clear and detailed technical documentation of the model and its conclusions should be made available. For instance, the only paper that differed from the others (Rumi et al., 2023) [26] used a different relative vaccine effectiveness assumption to estimate a key factor for cost-effectiveness, for which they offer an explanation. Such technical appendices allow the reader to examine the model structure, assumptions, and inputs carefully.

Moreover, this review only considered studies that were peer reviewed. Another review of adjuvanted trivalent influenza vaccines included conference abstracts to reduce publication bias [8]. However, we chose to exclude conference abstracts, as they may have incomplete, inaccurate, or preliminary results that are not appropriate for a systematic review. Finally, the review was not registered with PROSPERO. The rationale for registration is to avoid duplication of effort, foster collaboration, and minimise bias, as there is less room for selective reporting or outcome switching. However, the aim of this review was to provide timely and robust evidence on the cost-effectiveness of adjuvanted quadrivalent influenza vaccines. There are no caveats attached to the research question suggestive of a change in direction mid-review.

## 5. Conclusions

Information on the cost-effectiveness of aQIV among older persons is needed to inform reimbursement decisions. This study found ten papers that estimated the cost-effectiveness of aQIV for older persons against seasonal influenza. Nine papers, including those that deployed more sophisticated dynamic modelling techniques, concurred that aQIV was cost-effective or cost-saving relative to standard-dose QIV, high-dose QIV, and recombinant QIV. The one paper with contrary findings adopted a distinct modelling approach. While all models had limitations, based on the analyses presented, it is reasonable to conclude that there is a broad consensus as to the cost-effectiveness of aQIV in this population group. Further work in the area is warranted specifically to address gaps in the quality of data input into models and the transparency of models. Policymakers should also be alert to broader equity and efficiency considerations that may arise, especially where access is conditional on factors other than need.

## Figures and Tables

**Table 1:** Main characteristics of included studies.

Author, Year	Country, Currency (Year)	Population	Evaluation Type	Model Type	Perspective	Time Horizon	Discount Rate (per Annum)	Strategies	Uncertainty Analysis
Kohli et al., 2021 [17]	UK; UK Sterling (2020)	65–74 years; ≥75 years	CUA	SEIR + static decision tree	Payer	1 Influenza season	Costs and outcomes: 3.5%	aQIV v high dose QIV	DSA; PSA
Calabro et al., 2022 [18]	Italy; Euros (2020)	≥65 years (stratified into age groups)	CUA; CEA	SEIR + static decision tree	Payer; societal	1 Influenza season	Indirect costs and outcomes: 3%	aQIV v standard dose QIV	DSA; PSA
Fochesato et al., 2022 [19]	Spain; Euros (2021)	≥65 years	CUA	SEIR + static decision tree	Payer; societal	1 Influenza season	Costs: N/A; outcomes: 3%	aQIV v standard dose QIV	DSA; PSA
Kohli et al., 2022 [20]	Germany; Euro (2022)	65–74 years; ≥75 years	CUA	SEIR + static decision tree	Statutory health Insurance; societal	1 Influenza season	Costs and outcomes: 3%	aQIV v standard dose QIV; aQIV v high dose QIV	DSA; PSA
Ruiz-Aragon et al., 2022 [21]	Spain; Euro (2021)	65–69 years; 70+ years	CUA; CEA	Static decision tree	Payer; societal	1 Influenza season	Costs and outcomes: 3%	aQIV v high dose QIV	DSA; PSA
Jacob et al., 2023 [22]	Nodic; Euros (2022)	65–74 years; ≥75 years	CUA; CEA	Static decision tree	Payer; societal	1 Influenza season	Costs and outcomes: 3.5% Denmark; 4% Norway; 3% Sweden	aQIV v standard dose QIV; aQIV v high-dose QIV	DSA; PSA
Marbaix et al., 2023 [23]	Belgium; Euro (2023)	65–74 years; ≥75 years	CUA; CEA	Static decision tree	Payer	1 Influenza season	Costs: unclear; outcomes: 1.5%	aQIV v standard dose QIV; aQIV v high dose QIV	DSA; PSA
Nguyen et al., 2023 [24]	Ireland; Euro (2022)	65–74 years; ≥75 years	CUA	SEIR + static decision tree	Payer; societal	1 Influenza season	Costs: N/A; outcomes: 3%	aQIV v standard dose QIV	DSA; PSA
Ruiz-Aragon et al., 2023 [25]	Spain; Euro (2021)	65–69 years; ≥75 years	CUA	Static decision tree	Payer; societal	1 Influenza season	Costs and outcomes: 3%	aQIV v recombinant QIV	DSA; PSA
Rumi et al., 2023 [26]	Italy; Euro (2019)	65–74 years; ≥75 years	CUA	Static decision tree	Payer	1 Influenza season	Costs: N/A; outcomes: 3%	aQIV v high dose QIV	DSA; PSA

CEA: cost-effectiveness analysis; CUA: cost–utility analysis; SEIR: Susceptible–Exposed–Infectious–Removed; DSA: deterministic sensitivity analysis; PSA: probabilistic sensitivity analysis.

**Table 2:** Key assumptions and findings of included studies.

Author, Year, Country	Key Assumptions	Key Findings (Base Case)	Main Drivers of Cost-Effectiveness	Author Conclusions
Kohli et al., 2021 [17] United Kingdom	**Vaccine(s) compared to aQIV:** High-dose QIV **Price of vaccines:** aQIV: GBP 11.88 (list price) High-dose QIV: up to GBP 20.00 (list price) **Relative vaccine effectiveness:** aQIV vs. high-dose QIV: −2.5%, 3.2%, 8.9% **Vaccine coverage:** 68–80% (varying by age group) **Infuenza attack rate:** Not reported	**Burden of disease avoided:** None reported **ICER information:** For ICER to fall below GBP 20,000/QALY, unit price of high-dose QIV should be less than: GBP 12.94 (for rVE of −2.5%) GBP 10.44 (for rVE of 3.2%) GBP 7.67 (for rVE of 8.9%) ICER threshold: <GBP 30,000/QALY	**Deterministic sensitivity analysis findings:** No key drivers as relative vaccine effectiveness was not statistically different between vaccines. **Probabilistic sensitivity analysis findings:** Scenario analysis performed relating to cost given 3 levels of relative vaccine effectiveness.	**Conclusions for aQIV v high dose QIV** A small difference favoring aQIV when rVE was 3.2% or 8.9% or favouring high-dose QIV if rVE was −2.5%. Relative vaccine effectiveness in this patient population was not statistically significantly different between vaccines and aQIV was less costly.
Calabro et al. (2022) Italy [18]	**Vaccine(s) compared to aQIV:** Standard-dose QIV **Price of vaccines:** From HTA document (in Italian) **Relative vaccine effectiveness:** from HTA document (in Italian) aQIV vs. standard-dose QIV: 34.6% (2,66) **Vaccine coverage:** Taken from institutional reports; not reported in research paper **Influenza attack rate:** Not reported	**Burden of disease avoided:** Influenza cases avoided: 111,417 Hospitalizations avoided: 363 Deaths avoided: 195 **ICER information:** Payer ICER: EUR 14,441/QALY Societal ICER: EUR 11,748/QALY ICER threshold: <EUR 30,000/QALY	**Deterministic sensitivity analysis findings:** Total number of infections Vaccine efficacy Vaccine cost Probability of health seeking, death, and complications **Probabilistic sensitivity analysis findings:** aQIV cost-effective in 95% of iterations compared to standard-dose QIV	**Conclusions for** **aQIV vs. standard-dose QIV** From both a payer and a societal perspective, aQIV is cost-effective compared to standard-dose QIV in an older population at a willingness-to-pay threshold of EUR 30,000/QALY.
Fochesato et al. (2022) Spain [19]	**Vaccine(s) compared to aQIV:** Standard-dose QIV **Price of vaccines:** aQIV: EUR 13 Standard-dose QIV: EUR 9.50 **Relative vaccine effectiveness:** aQIV v standard dose QIV: 3.9% and 34.6% **Vaccine coverage:** 59.8–72.23% (varying by age) **Influenza attack rate:** Not reported	**Burden of disease avoided:** Complicated flu cases avoided: 43,664 Hospitalizations avoided: 111 Deaths avoided: 569 (for rVE of 34.6%) **ICER information:** Payer ICER: EUR 2240/QALY (for rVE of 34.6%) EUR 6694/QALY (for rVE of 13.9%) Societal ICER: cost saving (for rVE of 34.6%) EUR 3936/QALY (for rVE of 13.9%) ICER threshold: <EUR 25,000/QALY	**Deterministic sensitivity analysis findings:** Vaccine efficiency Vaccine cost Vaccine coverage **Probabilistic sensitivity analysis findings:** aQIV is cost-effective in 65% of iterations compared to standard-dose QIV (where rVE is 34.6%) and in 52.4% of iterations (where rVE is 13.9%).	**Conclusions for** **aQIV v standard dose QIV** From both a payer and a societal perspective, aQIV is cost-effective compared to standard-dose QIV in an older population at a willingness-to-pay threshold of EUR 30,000/QALY.
Kohli et al., 2022 [20] Germany	**Vaccine(s) compared to aQIV:** Standard-dose QIV High-dose QIV **Price of vaccines:** aQIV: EUR 19.21 (reimbursed price) standard dose QIV: EUR 12.56 (reimbursed price) **Relative vaccine effectiveness:** aQIV vs. standard-dose QIV: 13.9% (4.2, 23.5) aQIV vs. high-dose QIV: 3.2% (−2.5, 8.9) **Vaccine coverage:** 40% **Influenza attack rate:** Not reported	**For aQIV v standard dose QIV** **Burden of disease avoided:** Medical cases avoided: 38,755 Hospitalisations avoided: 476 Deaths avoided: 287 **ICER information:** **Payer ICER:** EUR 20,000/QALY (2 severe seasons) **Societal ICER:** EUR 17,200/QALY (2 severe seasons **ICER threshold:** <EUR 25,000/QALY–EUR 50,000/QALY **For aQIV vs. high-dose QIV** **Burden of disease avoided:** Medical cases avoided: 30,897 Hospitalizations avoided: 380 Deaths avoided: 229 **ICER information:** Cost-saving; aQIV dominated high-dose QIV	**Deterministic sensitivity analysis findings:** Vaccine coverage Rate of hospitalisation for flu Vaccine coverage **Probabilistic sensitivity analysis findings:** Probability of CE varied depending on the willingness-to-pay threshold used (varied from EUR 20,000/QALY to EUR 50,000/QALY. Results of PSA not reported in text but available visually in Supplementary Materials.	**Conclusions for aQIV vs. standard-dose QIV and high-dose QIV** Both of the enhanced vaccines reduced the number of influenza cases, hospitalisations, and deaths in the German population compared to standard-dose QIV. As aQIV was the most cost-effective vaccine in the base case, the use of this vaccine in the oldest age group resulted in the lowest amount of disease. The difference between the three vaccines was most pronounced as the average number of severe influenza seasons increased.
Ruiz-Aragon et al., 2022 [21] Spain	**Vaccine(s) compared to aQIV:** High-dose QIV **Price of vaccines:** aQIV: EUR 13 (list price) High-dose QIV: EUR 25 (list price) **Relative vaccine effectiveness:** aTIV vs. high-dose TIV: 4% (−0.05, 8.4) **Vaccine coverage:** 54.7% **Influenza attack rate:** Not reported	**Burden of disease avoided:** Symptomatic cases avoided: 5405 Primary care visits avoided: 760 Hospitalisations avoided: 442 Emergency room visits: 171 Deaths avoided: 26 **ICER information:** aQIV dominates high-dose QIV, as it is more expensive and less effective. Payer perspective: savings of EUR 63.6 M Societal perspective: savings of EUR 64.2 M ICER threshold: <EUR 25,000/QALY	**Deterministic sensitivity analysis findings:** Vaccine cost Vaccine coverage Relative vaccine effectiveness **Probabilistic sensitivity analysis findings:** aQIV was cost-effective in 100% of iterations and dominant in 96% of iterations.	**Conclusions for aQIV vs. high-dose QIV** aQIV is cost-saving compared to high-dose QIV from both a payer and a societal perspective. This finding was driven by the similarity in vaccine effectiveness and lower cost of aQIV.
Jacob et al., 2023 [22] Denmark, Norway, Sweden	**Vaccine(s) compared to aQIV:** Standard-dose QIV High-dose QIV **Price of vaccines:** QIV: EUR 9.10–EUR 11.00 (by jurisdiction) Price of aQIV: 170–189% of price of QIV Price of high-dose QIV: EUR 25 **Relative vaccine effectiveness:** aQIV vs. high-dose QIV: 3.2% (−2.5, 8.9) **Vaccine coverage:** 60–75% (varying by jurisdiction) **Influenza attack rate:** 7.2%	**Denmark:** **Burden of disease avoided:** Symptomatic cases avoided: 6238 GP cases avoided: 1871 Hospitalizations avoided: 307 Deaths avoided: 54 **ICER information:** Payer ICER: EUR 10,170/QALY Societal ICER: EUR 5472/QALY ICER threshold: <EUR 30,000/QALY **Norway:** **Burden of disease avoided:** Symptomatic cases avoided: 3810 GP cases avoided: 1143 Hospitalizations avoided: 187 Deaths avoided: 32 **ICER information:** Payer ICER: EUR 12,515/QALY Societal ICER: EUR 7906/QALY ICER threshold: <EUR 30,000/QALY **Sweden:** **Burden of disease avoided:** Symptomatic cases avoided: 8724 GP cases avoided: 2617 Hospitalizations avoided: 431 Deaths avoided: 75 **ICER information:** Payer ICER: EUR 9894/QALY Societal ICER: EUR 4856/QALY ICER threshold: <EUR 30,000/QALY **aQIV vs. high-dose QIV** aQIV vs. high-dose QIV would be cost-saving if expanded to the elderly population with savings of EUR 7.1 M in Denmark, EUR 4.7 M in Norway and EUR 8.5 M in Sweden.	**Deterministic sensitivity analysis findings:** aQIV v standard dose QIV Relative vaccine efficiency Cost ratio Influenza attack rate Vaccine cost **Probabilistic sensitivity analysis findings:** From the payer perspective, aQIV is likely to be more cost-effective than standard-dose QIV in 90% of iterations in Denmark, 77% of iterations in Norway, and 75% of iterations in Sweden.	**Conclusions for** **aQIV v standard dose QIV** aQIV was cost-effective compared to standard dose QIV in Denmark, Norway and Sweden at a willingness to pay threshold of EUR 30,000/QALY. **Conclusions for aQIV vs. high-dose QIV** aQIV was cost-saving (cummulative savings of over EUR 20 M across Denmark, Norway, and Sweden).
Marbaix et al., 2023 [23] Belgium	**Vaccine(s) compared to aQIV:** Standard-dose QIV High-dose QIV **Price of vaccines:** aQIV: EUR 24.73 (reimbursement price) Standard dose QIV: EUR 12.94 (reimbursement price) High-dose QIV: EUR 32.62 (reimbursement price) **Relative vaccine effectiveness:** aQIV vs. standard-dose QIV: - aQIV vs. high-dose QIV: −2.5%, 3.2%, 8.9% (assumed equivalence of TIV and QIV) **Vaccine coverage:** 62% **Influenza attack rate:** 5% (in vaccinated older adults)	**aQIV vs. standard-dose QIV** **Burden of disease avoided:** Hospitalizations avoided: 530 Deaths avoided: 66 Life years saved: 656 QALYs gained: 451 **ICER information:** Payer ICER: EUR 15,227/QALY Societal ICER: not calculated ICER threshold: <EUR 35,000/QALY **aQIV vs. high-dose QIV** aQIV was dominant alternative, because aQIV is expected to be less expensive and slightly more effective or at least as effective.	**Deterministic sensitivity analysis findings:** Vaccine cost Relative vaccine effectiveness Influenza attack rate Vaccine coverage **Probabilistic sensitivity analysis findings:** The probability of aQIV being cost-effective was estimated to be 82% at a willingness-to-pay threshold of EUR 35,000/QALY.	**Conclusions for aQIV vs. standard-dose QIV** aQIV is cost-effective compared to standard-dose QIV in this patient population, with an incremental cost-effectiveness ratio of EUR 15,227/QALY from the payer perspective. **Conclusions for aQIV vs. high-dose QIV** aQIV is cost-saving (dominant) compared to high-dose QIV, as the difference in relative effectiveness is small and aQIV is less costly.
Nguyen et al., 2023 [24] Ireland	**Vaccine(s) compared to aQIV:** Standard dose **Price of vaccines:** aQIV: EUR 18 (market price) standard-dose QIV: eur 10 (market price) **Relative vaccine effectiveness:** aQIV vs. standard-dose QIV: 13.9% (3,24) **Vaccine coverage:** 68–80% (varying by age group and risk) **Influenza attack rate:** 7–14% (by age group)	**Burden of disease avoided:** for aQIV v standard-dose QIV Symptomatic cases avoided: 4107 Hospitalisations avoided: 156 Deaths avoided: 42 **ICER information:** Payer ICER: EUR 12,970/QALY Societal ICER: EUR 2420/QALY ICER threshold: <EUR 45,000/QALY	**Deterministic sensitivity analysis findings:** Relative vaccine effectiveness Vaccine cost Disease incidence **Probabilistic sensitivity analysis findings:** Not presented	**aQIV vs. standard-dose QIV** aQIV is highly cost-effective compared to standard-dose QIV in an elderly population, with an ICER of EUR 12,970/QALY from the payer perspective and EUR 2420/QALY from a societal perspective. This is below the below the ICER threshold of EUR 45,000/QALY in Ireland.
Ruiz-Aragon et al., 2023 [25] Spain	**Vaccine(s) compared to aQIV:** Recombinant QIV **Price of vaccines:** aQIV: EUR 13 (tender price) Recombinant QIV: EUR 25 (tender price) **Relative vaccine effectiveness:** **QIV vs. aTIV:** 10.7% (2.7, 17.9) **Vaccine coverage:** 69.4% **Influenza attack rate:** Not reported	**Burden of disease avoided:** Not presented **ICER information:** **Payer ICER:** EUR 101,612/QALY **ICER threshold:** <EUR 25,000/QALY	**Deterministic sensitivity analysis findings:** Vaccine cost Vaccine coverage **Probabilistic sensitivity analysis findings:** aQIV was more cost-effective than recombinant QIV in 99.7% of simulations.	**aQIV vs. recombinant QIV** Recombinant QIV is not CE for older persons compared to aQIV; to be cost-effective the relative vaccine effectiveness of recombinant QIV v aQIV needs to be 34.1%.
Rumi et al., 2023 [26] Italy	**Vaccine(s) compared to aQIV:** High-dose QIV **Price of vaccines:** **aQIV:** EUR 15.45 **High-dose QIV:** EUR 32.27 **Relative vaccine effectiveness:** aQIV vs. standard-dose QIV: 0% (0–20%) high-dose QIV vs. standard dose QIV: 18.2%, 24.2% **Vaccine coverage:** 54.4% (no range given) **Influenza attack rate:** 7.2% (5.8–8.6%)	**Burden of disease avoided:** Not presented **ICER information:** Payer ICER: EUR 9805 (where relative efficacy of aQIV vs. standard dose QIV was assumed to be 6%) Societal ICER: not assessed ICER threshold: <EUR 30,000/QALY	**Deterministic sensitivity analysis findings:** Efficacy of high-dose QIV vs. standard-dose QIV Relative efficacy of aQIV vs. standard-dose QIV Vaccine cost **Probabilistic sensitivity analysis findings:** The cost effectiveness acceptability curve showed that high-dose QIV had a high probability (97%) of being cost-effective compared to aQIV at a WTP threshold of EUR 30,000 per QALY gained.	**Switch from aQIV to high-dose QIV** Switching to high-dose QIV from aQIV would be cost-effective (or cost-saving in a scenario where hospitalisation was “possibly” related to influenza).

QALY: Quality-adjusted life year; LY: life year; rVE: relative vaccine effectiveness; ICER: incremental cost-effectiveness ratio; HTA: Health Technology Assessment; GP: general practitioner; WTP: willingness to pay.

## Supplementary Materials

The following supporting information can be downloaded at: https://www.mdpi.com/article/10.3390/vaccines12050523/s1, Table S1: PICO search strategy; Table S1: CHEERS (2022) checklist; Table S3: Costs updated to EUR €2023.
