# Supplementary material for: Use of Adjuvanted Quadrivalent Influenza Vaccine in Older-Age Adults: A Systematic Review of Economic Evidence
Source: Vaccines (Basel). 2024 May 10;12(5):523. doi: 10.3390/vaccines12050523 (PMC11126004; doi:10.3390/vaccines12050523)
Supplement: Supplementary file 1 [file vaccines-12-00523-s001.zip › vaccines-2935495-supplementary.pdf]

Table S1: PICO search strategy

		**PICO** [Pubmed]
**P**		"older adults" [tiab] OR<br>“older adult” [tiab] OR<br>Elderly [tiab] OR<br>senior* [tiab] OR<br>"aged 65+" [tiab] OR<br>geriatric* [tiab] OR<br>“aged 65” [tiab] OR<br>“aged ≥65 years” [tiab] OR<br>“aged 65+” [tiab] OR<br>“≥65” [tiab] OR<br>“65+" [tiab]
		**AND**
**I**		“aQIV” [tiab] OR<br>“Fluad” [tiab] OR<br>“MF59”[tiab] OR<br>Adjuvanted [tiab] OR<br>“MF59-adjuvanted quadrivalent influenza vaccine”[tiab] OR<br>“Adjuvanted quadrivalent influenza vaccine”[tiab] OR<br>“Quadrivalent adjuvanted influenza vaccine”[tiab] OR<br>“adjuvanted quadrivalent”[tiab] OR<br>“quadrivalent adjuvanted”[tiab] OR<br>“QIV-AD”[tiab]
		**AND**
**C**		Comparator can be no vaccine, standard dose QIV or high dose TIV or QIV. These terms are not explicitly used in the search strategy because of the number of hits they generate. However, the term adjuvanted and QIV and CEA (which by definition is comparative will capture relevant comparators).
		**AND**
**O**		Cost* [tiab] OR<br>Econ* [tiab] OR<br>“Cost-effectiveness” [tiab] OR<br>“Cost-utility” [tiab] OR<br>“Cost-benefit” [tiab] OR<br>“Economic evaluation” [tiab] OR

		"Economic analysis" [tiab] OR<br>"Health technology assessment"[tiab] OR<br>HTA [tiab] OR<br>"Budget impact analysis" [tiab] OR<br>BIA [tiab] OR<br>Model [tiab] OR<br>Markov [tiab]
**LIMIT**		Human, English language, 2013-present

Table S2: Cheers (2022) checklist

CHEERS (2022) Checklist*		Calabro et al. 2022	Fochesato et al. 2022	Jacob et al. 2023	Kohli et al., 2021	Kohli et al., 2022	Marbaix et al. 2023	Nguyen et al., 2023	Ruiz-aragon et al. 2022	Ruiz-aragon et al. 2023	Rumi et al. 2023
1	**Title:** Identify the study as an economic evaluation and specify the interventions being compared.	1	1	2	4603	1	608	1	1	1	1
2	**Abstract:** provide a structured summary that highlights context, key methods, results, and alternative analyses	1	1	1	4603	1	608	1	1	1	1
3	**Background and objectives:** give the context for the study, the study question, and its practical relevance for decision making in policy or practice.	2	1-2	1-2	4603	1-2	608 to 609	1-2	1-2	1-2	2
4	**Methods:** indicate whether a health economic analysis plan was developed and where available.	No	No	No	No	No	No	No	No	No	No
5	**Study population:** describe characteristics of the study population (such as age range, demographics, socioeconomic, or clinical characteristics).	3**	2**	2**	4603**	2**	609**	2**	2**	2**	2**
6	**Setting and location:** provide relevant contextual information that may influence findings.	3	3	3-4	4604	2	609	2	2	2	2

7	**Comparators:** describe the interventions or strategies being compared and why chosen.	1	2	3	4603	2	609	2	2	2	2
8	**Study perspective:** state the perspective(s) adopted by the study and why chosen	4	2	3	4604	6	610	3	2	3-4	2
9	**Time horizon:** state the time horizon for the study and why appropriate.	4	7**	3	4604	6	610	unclear	2	2	2
10	**Discount rate:** report the discount rate(s) and reason chosen.	4	8**	3	4604	2	610	4	2	3	2
11	**Selection of outcomes:** describe what outcomes were used as the measure(s) of benefit(s) and harm(s).	4 (and Figure 1)	8	6	4604	2	612	4	2	3	2
12	**Measurement of outcomes:** describe how outcomes used to capture benefit(s) and harm(s) were measured.	4	8	6	4604	6	610	4	4	4	3
13	**Valuation of outcomes:** describe the population and methods used to measure and value outcomes.	No	8	6	4604	6	610	?	4-5	4	3
14	**Measurement and valuation of resources and costs:** describe how costs were valued.	No	7	6	4604	6	610	3-4	5	4	3

15	**Currency, price and date conversion:** report the dates of the estimated resource quantities and unit costs, plus the currency and year of conversion.	No	No	No	4604	6**	610	unclear	5	4	3
16	**Rationale and description of model:** If modeling is used, describe in detail and why used. Report if the model is publicly available and where it can be accessed.	No	3**	2**	4604**	4**	609**	2, 3**	2**	2**	3
17	**Analytics and assumptions:** describe any methods for analysing or statistically transforming data, any extrapolation methods, and approaches for validating any model used.	No	No	No	none	none	none	2**	none	None	None
18	**Characterising heterogeneity:** describe any methods used for estimating how the results of the study vary for subgroups.	No	No	No	None	None	None	None	None	None	None
19	**Characterising distributional effects:** describe how impacts are distributed across different individuals or adjustments made to reflect priority populations.	No	No	No	None	None	None	None	None	None	None
20	**Characterising uncertainty:** describe methods to characterize any sources of uncertainty in the analysis.	No	8	8	4605 and 4606	7-11	613-615	5-7	5	5	5

21	**PPI:** Describe any approaches to engage patients or service recipients, the general public, communities, or stakeholders (such as clinicians or payers) in the design of the study.	No	No	No	None	None	None	None	None	None	None
22	**Study parameters:** report all analytic inputs (such as values, ranges, references) including uncertainty or distributional assumptions.	No	3-10**	4-10**	4604-4605 **	5**	613**	3-5**	3-6**	3-5**	2 to 4
23	**Summary of main results:** report the mean values for the main categories of costs and outcomes of interest and summarize them in the most appropriate overall measure.	No	7	9 and 10	4605 and 4606 **	9	613	5	6	5	5
24	**Effects of uncertainty:** describe how uncertainty about analytic judgments, inputs, or projections affect findings. Report the effect of choice of discount rate and time horizon, if applicable.	7**	10**	11-12 **	4605-4606 **	10, 11 **	613-615 **	5-7**	7-8**	5-6**	5 and 6
25	**Effect of PPI:** report on any difference patient/service recipient, general public, community, or stakeholder involvement made to the approach or findings of the study	No	No	No	None	None	None	None	None	None	None

26	**Study findings, limitations, generalisability and current knowledge:** report key findings, limitations, ethical or equity considerations not captured, and how these could affect patients, policy, or practice.	10 (partially)	11-12	7 to 13	4609	11-13	616	8-9	8	6-8	4 to 7
27	**Source of funding:** describe how the study was funded and any role of the funder in the identification, design, conduct, and reporting of the analysis	10	12	15	4609	13	617	9	9	9	7
28	**Conflicts of interest:** report authors conflicts of interest according to journal or International Committee of Medical Journal Editors requirements.	10	12	15	4609	13	617	9	9	9	9

**The numbers in the table refer to the page number on which the item was reported in the associated research paper*

Table S3: Costs updated to EUR €2023

Author, year	Country, currency (year)	Original costs	Updated costs €2023
Calabro et al. 2022	Italy; EUR €2020	Payer ICER: €14,441/QALY<br>Societal ICER: €11,748/QALY	Payer ICER: €15,632/QALY<br>Societal ICER: €12,717/QALY
Fochesato et al. 2022	Spain; EUR €2021	Payer ICER:<br>€2,240/QALY (for rVE of 34.6%)	€2,458/QALY (for rVE of 34.6%)

		€6,694/QALY (for rVE of 13.9%)<br>Societal ICER:<br>Cost saving (for rVE of 34.6%)<br>€3,936/QALY (for rVE of 13.9%)	€7,347/QALY (for rVE of 13.9%)<br>Societal ICER:<br>Cost saving (for rVE of 34.6%)<br>€4,320/QALY (for rVE of 13.9%)
Jacob et al. 2023	Nodic; EUR €2022	**Denmark:**<br>Payer ICER: €10,170/QALY<br>Societal ICER: €5,472/QALY<br>**Norway:**<br>Payer ICER: €12,515 /QALY<br>Societal ICER: €7,906 /QALY<br>**Sweden:**<br>Payer ICER: €9,894 /QALY<br>Societal ICER: €4,856/QALY	**Denmark:**<br>Payer ICER: €10,156/QALY<br>Societal ICER: €5,464/QALY<br>**Norway:**<br>Payer ICER: €12,516/QALY<br>Societal ICER: €7,906/QALY<br>**Sweden:**<br>Payer ICER: €10,540 /QALY<br>Societal ICER: €5,173/QALY
Kohli et al. 2021	UK; GBP £2020*	£12.94 (for rVE of -2.5%)<br>£10.44 (for rVE of 3.2%)<br>£7.67 (for rVE of 8.9%)	€16.91 (for rVE of -2.5%)<br>€13.65 (for rVE of 3.2%)<br>€10.03 (for rVE of 8.9%)
Kohli et al. 2022	Germany; EUR €2022	Payer ICER: €20,000/QALY<br>Societal ICER: €17,200/QALY	Payer ICER: €21,111/QALY<br>Societal ICER: €18,156/QALY
Marbaix et al. 2023	Belgium; EUR €2023	Payer ICER: €15,227/QALY<br>Societal ICER: not calculated	Payer ICER: €15,227/QALY<br>Societal ICER: not calculated

Nguyen et al. 2023	Ireland; EUR €2022	Payer ICER: €12,970/QALY<br><br>Societal ICER: €2,420/QALY	Payer ICER: €13,605/QALY<br><br>Societal ICER: €2,538/QALY
Ruiz-Aragon et al. 2022	Spain; EUR €2021	saving of €63.6 M<br><br>saving of €64.2 M<br><br>€101,612/QALY	saving of €67.0 M<br><br>saving of €67.7 M<br><br>€107,082/QALY
Ruiz-Aragon et al. 2023	Spain; EUR €2021	Payer perspective:<br><br>saving of €63.6 M<br><br>Societal perspective:<br><br>saving of €64.2 M	Payer perspective:<br><br>saving of €69.8 M<br><br>Societal perspective:<br><br>saving of €70.5 M
Rumi et al. 2023	Italy; EUR €2019	Payer ICER: €9,805 (where relative efficacy of aQIV vs standard dose QIV was assumed as 6%)	Payer ICER: €10,781 (where relative efficacy of aQIV vs standard dose QIV was assumed as 6%)

*Updated from £2020 to €2023 at exchange rate of £1=€1.18 (16/04/24); rVE=relative vaccine effectiveness

*Conversion tool: https://eppi.ioe.ac.uk/costconversion/default.aspx
